# The relationship between parental stress and the mediating effect of organizational support perceived by nurses: a cross-sectional study

**DOI:** 10.1590/1980-220X-REEUSP-2025-0162en

**Published:** 2025-12-05

**Authors:** Na Liu, Lihua Niu, Qian Hu, Yan Wang, Donglian Zheng, Miao Yao, Guangli Mi

**Affiliations:** 1 Ningxia Medical University, General Hospital, Yinchuan, China.

**Keywords:** Organizational Culture, Nurses, Male, Parenting, Negotiating, Job Satisfaction, Cultura Organizacional, Enfermeiros, Poder Familiar, Negociação, Satisfação no Emprego

## Abstract

**Objective:**

Examine the relationship between parenting stress and thriving at work among nurses. Examine whether organizational support mediates the association between parenting stress and thriving at work among nurses.

**Methods:**

A questionnaire survey was conducted with 534 clinical nurses using the Parenting Stress Scale for Clinical Nurses, the Thriving at Work Scale, and the Perceived Organizational Support Questionnaire.

**Results:**

The mean total scores for nursing parenting stress, perceived organizational support, and the Thriving at Work Scale were 2.72 ± 0.47, 3.63 ± 0.81, and 4.80 ± 0.74, respectively. A significant negative correlation was observed between nursing parenting stress and thriving at work (r_1_ = −0.233, p < 0.001) and between nursing parenting stress and perceived organizational support (r_2_ = −0.289, p < 0.001). A significant positive correlation was also observed between perceived organizational support and thriving at work (r_3_ = 0.469, p < 0.001). Perceived organizational support partially mediated the relationship between parenting stress and thriving at work, accounting for 52.72% of the total effect.

**Conclusion:**

This study supports the mediating role of perceived organizational support in the relationship between parenting stress and thriving at work among nurses. Interventions to promote perceived organizational support among nurses with parenting stress should be studied to improve their well-being.

## RESUMO

**Objetivo:**

Examinar a relação entre o estresse parental e o bem-estar no trabalho entre enfermeiros e enfermeiras, e examinar se o apoio organizacional medeia a associação entre o estresse parental e o bem-estar entre enfermeiros e enfermeiras.

**Métodos:**

Foi realizada uma pesquisa por questionário com 534 enfermeiros e enfermeiras clínicos, utilizando a Escala de Estresse Parental para Enfermeiros e Enfermeiras Clínicos, a Escala de Bem-estar no Trabalho e o Questionário de Apoio Organizacional Percebido.

**Resultados:**

As pontuações totais do estresse parental dos enfermeiros, do apoio organizacional percebido pelos enfermeiros e da Escala de Prosperidade no Trabalho foram (2,72 ± 0,47), (3,63 ± 0,81) e (4,80 ± 0,74), respectivamente. Foi observada uma correlação negativa significativa entre o estresse parental de enfermagem e o sucesso no trabalho (r1 = −0,233, P < 0,001) e o apoio organizacional percebido pelos enfermeiros (r2 = −0,289, P < 0,001), bem como uma correlação positiva significativa entre o apoio organizacional percebido pelos enfermeiros e o sucesso no trabalho (r3 = 0,469, P < 0,001). O apoio organizacional percebido pelos enfermeiros mostrou um efeito mediador parcial na relação entre o estresse parental dos enfermeiros e o sucesso no trabalho, representando 52,72% do efeito total.

**Conclusão:**

Este estudo apoia um papel mediador do apoio organizacional percebido pelos enfermeiros na relação entre o estresse parental dos enfermeiros e o sucesso no trabalho. Intervenções para promover o apoio organizacional percebido pelos enfermeiros devem ser estudadas para o sucesso no trabalho de enfermeiros com estresse parental.

## INTRODUCTION

As positive psychology and organizational behavior have developed, an increasing number of researchers have studied ways to enhance nurses’ enthusiasm and foster the mutual growth of nurses and organizations through positive psychological experiences. Despite the heavy workload of nursing, some nurses may experience fatigue due to an inability to cope with work pressure. However, many nurses persevere in their roles and experience positive growth. Previous studies have indicated that nurses may experience negative emotions, such as burnout and fatigue, as well as positive emotions, such as long-term work^(1, 2)^ growth and in their happiness^(3, 4)^.

The concept of “thriving” at work was first proposed in 2005^(5)^ and is defined as a positive psychological state in which individuals experience learning and vitality at work. Research shows that thriving at work enhances adaptability^(6)^, improves performance, reduces burnout, and promotes health^(7)^.

In China, nurses are generally younger, with over 60% of nursing professionals being under 35 years old, and most of them are women^(8)^. Female nurses of childbearing age play a pivotal role in their departments. They face professional pressures such as high labor intensity, irregular work schedules, long-term mental stress, and pressures related to promotion, scientific research, and teaching^(9, 10)^. Meanwhile, since the implementation of the “two-child, three-child” policy, attention has focused on the fertility issues and childbearing challenges faced by nurses of childbearing age. Nurses returning to work after childbirth often feel unfamiliar with clinical practices and may experience a disconnect from clinical work. Additionally, raising children requires considerable time and energy, which leads to increased work stress. Many nurses report that “childcare is stressful”^(11,12,13)^. The number of children correlates positively with nurses’ turnover^(14)^, and the turnover rate of female nurses who gave birth to a second child is as high as 62.73%^(15)^. Nurses with parenting responsibilities are particularly susceptible to psychological and physical strain, including anxiety, fatigue, and occupational burnout. These issues are often manifested through sleep deprivation and a heightened emotional burden. These challenges can diminish work engagement and increase the likelihood of turnover. Such exhaustion undermines nurses’ well-being, compromises the quality of care, and raises the risk of clinical errors, thereby endangering patient safety. Furthermore, parenting-related stress can lead to increased absenteeism and resignations, exacerbating staffing shortages, this intensifies the workload of remaining staff and ultimately reduces overall operational efficiency. These issues present substantial obstacles to the stable and sustainable functioning of healthcare institutions. Therefore, nursing managers must pay close attention to the stress clinical nurses experience related to raising children and reducing nurse turnover.

According to the job requirements–resources model, individuals need resources from others or organizations to cope with stress^(16)^. Perceived organizational support (POS) refers to whether employees feel their contributions are valued and whether they are cared for. A high sense of organizational support can provide employees with positive emotional support, mitigate the negative effects of stress, help them maintain an active state, both physically and psychologically, enhance job prosperity^(17)^, and reduce their desire to leave^(18, 19)^. Domestic and international scholars have primarily focused on thriving at work among nurses and the relationship between thriving at work, job crafting, and missed nursing care. However, the mediating mechanisms between parenting stress, thriving at work, and organizational support remain unclear.

Therefore, this study aims to examine the following: 1) the situation of clinical nurses’ parenting stress, organizational support, and thriving at work; 2) the relationship between parenting stress, perceived organizational support, and thriving at work among Chinese nurses; and 3) whether organizational support mediates the association between parenting stress and thriving at work.

## METHOD

### Ethical Consideration

This study was approved by the General Hospital of Ningxia Medical University (KYLL-2024-0054). The cover page of the questionnaire clearly explained the purpose of the study and assured participants of their right to anonymity and confidentiality, as well as their right to refuse participation. Informed consent was obtained from all participants.

### Study Design

This study employed a quantitative, cross-sectional design. The report adhered to the Strengthening the Reporting of Observational Studies in Epidemiology (STROBE) guidelines.

### Participants

Study participants were recruited from a general hospital in Ningxia, China, using convenience sampling. Those who met the following four inclusion criteria were invited to participate in the survey: 1) aged 22–45 years, 2) possessing a nurse’s qualification certificate, 3) being patients rather than relatives accompanying patients, 4) being registered as on-the-job nurses, and 5) providing informed consent and voluntarily participating. We excluded probationary and intern nurses, advanced nurses, and off-the-job nurses, such as those on sick leave, studying, or out for further study. We contacted Mandarin Chinese–speaking nurses working in the hospital face-to-face to identify those who met the inclusion criteria. We explained the purpose of the survey to them and asked them to participate as scheduled.

The survey was conducted from December 15, 2023, to February 19, 2024. The questionnaire was administered via Wenjuanxing (https://www.wjx.cn/), the most popular web-based questionnaire platform in China. Participants filled out the questionnaire online. Only questionnaires with answers to all question items were considered valid according to our predefined validation criterion. On August 20, 2022, we downloaded the returned questionnaires in Excel format from Wenjuanxing. A total of 534 questionnaires were returned, resulting in a response rate of 97.9% (534/545). We double-checked the returned questionnaires and found them all to be valid.

### Instruments

#### Dependent Variables

We used the Thriving at Work Scale^(20)^ to assess thriving at work among nurses. Developed at Georgetown University in the United States in 2012, the scale has since been translated into Chinese^(21, 22)^. The scale comprises ten items distributed across two dimensions: learning (five items) and vitality (five items). We employed a 7-point Likert scale ranging from “strongly disagree” to “strongly agree,” with items 4 and 8 being reverse-scored. Total scores can range from 10 to 70, with higher scores indicating a greater level of work prosperity. The Cronbach’s α coefficient for this scale was 0.822.

#### Independent Variable

We used the Parenting Stress Scale to evaluate the parenting stress experienced by nurses. Developed in Korea in 2021^(23)^, this scale was subsequently translated into Chinese^(24)^ and used to assess the parenting stress of nurses. The scale comprises 19 items categorized into four dimensions: psychological burden (six items), physical and mental fatigue (five items), shift work (five items), and working environment (three items). Respondents were asked to indicate their level of agreement with each item on a 4-point Likert scale ranging from “strongly disagree” to “strongly agree.” The total score ranges from 19 to 76 points. A higher score indicates greater parenting stress experienced by nurses. The total Cronbach’s α coefficient for this scale is 0.86, with coefficients for the four dimensions ranging from 0.81 to 0.86.

#### Mediator

We used the Perceived Organizational Support Questionnaire to evaluate the level of organizational support experienced by nurses. This scale was developed to measure the degree of organizational support for clinical nurses^(25)^. It is a single-dimensional scale comprising 15 items. We used a 5-point Likert scale ranging from “strongly disagree” to “strongly agree,” assigning scores from 1 to 5 in sequential order. Total scores range from 15 to 75, with higher scores indicating a greater sense of organizational support. The questionnaire’s content validity index is 0.940, and its Cronbach’s α coefficient is 0.953.

#### Covariates

The analyses included the following covariates: demographic and work-related characteristics, including sex, age, educational background, professional title, years of experience, employment method, department, position, number of night shifts per month, working hours per week, frequency of overtime per week, and whether the department provided additional care.

#### Statistical Analysis

We described demographic and work-related characteristics using frequencies, proportions, means, and standard deviations (SDs). Skewness and kurtosis tests were used to test the normality of the distribution of scores on the four scales. Pearson’s correlation analysis was used to explore the relationships among nurses’ parenting stress, organizational support, and thriving at work. A mediating effects model of nurses’ parenting stress, organizational support, and thriving at work was constructed using AMOS 23.0. SPSS 4.0 process macros tested the mediating effects of organizational support on the relationship between parenting stress and thriving at work. The percentile bootstrap method with deviation correction was used to test the significance of the intermediary effect. If the 95% confidence interval (CI) did not include zero, then the mediating effect was considered significant. Demographic and work-related characteristics were included as covariates in this model. Statistical significance was determined at a p-value of less than 0.05 (two-tailed).

## RESULT

### Descriptive Statistics

#### Sociodemographic Characterization of Nurses

This study examined the sociodemographic characteristics of 534 nurses in China in 2023 (Table 1). The results revealed that the nursing workforce was predominantly female (94.0%) and that the largest age group was 35–45 years old (37.8%). Regarding education, most held a bachelor’s degree (79.8%), while only 2.2% held a master’s degree or higher. Professional titles were primarily junior (55.8%) and intermediate (37.6%), and most nurses were non-permanent staff (75.5%). Work experience was relatively evenly distributed, with the highest proportion having six to 15 years of service (53.7% combined). Most (76.0%) held no managerial positions, and the most common departments were internal medicine (31.3%) and surgery (20.0%). Regarding night shifts, 33.1% of nurses did not work them, while 30.5% worked five to nine per month. Regarding working hours, 63.1% worked over 40 hours per week, and 41.0% sometimes worked overtime.

**Table 1:** Sociodemographic characterization of nurses (n = 534) – China, 2023.

Items	Category	Quantity	Percentage (%)
Gender	Male	32	6.0
	Female	502	94.0
Age (years)	22~29	162	30.4
	30~34	170	31.8
	35~45	202	37.8
Official academic credentials	College degree or below	96	19.0
	Undergraduate	426	79.8
	Graduate students and above	12	2.2
Professional title	Primary	298	55.8
	Middle rank	201	37.6
	Deputy senior and above	35	6.6
Employment mode	Not editing	403	75.5
	Officially editing	131	24.5
Working life	≤5	131	24.5
	6~10	146	27.3
	11~15	141	26.4
	16~20	58	10.9
	>20	58	10.9
Position	Without	406	76.0
	Teaching leader or responsible leader	89	16.7
	Head nurse	39	7.3
Department	Internal medicine	167	31.3
	Surgery	107	20.0
	Gynecology and obstetrics	26	4.9
	Pediatrics	35	6.6
	Intensive care unit	29	5.4
	Emergency department	62	11.6
	Operating room	28	5.2
	Other	80	15.0
Night shift per month	No night shift	177	33.1
	≤4	96	18.0
	5~9	163	30.5
	≥10	98	18.4
Working hours per week (hours)	<40	75	14.0
	40	122	22.8
	>40	337	63.1
Frequency of overtime work per week	Almost never	71	13.3
	Rarely	151	28.3
	Sometimes	219	41.0
	Often	93	17.4

#### Current Situation of Parenting Stress, Organizational Support, and Thriving at Work Among Clinical Nurses

The results revealed an overall parenting stress score of 2.72 ± 0.47 among clinical nurses (Table 2). The highest subscale score was for shift work (2.89 ± 0.60), followed by work environment (2.82 ± 0.63), psychological burden (2.64 ± 0.54), and physical-mental fatigue (2.59 ± 0.65). Regarding thriving at work, the total score was 4.80 ± 0.74; the “learning” dimension scored lower than the “vitality” dimension. Perceived organizational support scored 3.63 ± 0.81.

**Table 2:** The current situation of parenting stress, organizational support, and thriving among clinical nurses (N = 534) – China, 2023.

Items	Score range	Aggregate score	Average entry
**Parenting pressure of clinical nurses**	19~76	51.75±9.00	2.72±0.47
Psychological burden	4~24	15.83±3.27	2.64±0.54
Physical and mental fatigue	5~20	12.98±3.25	2.59±0.65
Shift work	5~20	14.45±3.02	2.89±0.60
Working environment	3~12	8.47±1.91	2.82±0.63
**Work prosperity**	14~70	48.06±7.43	4.80±0.74
Study	7~35	24.59±4.02	4.91±0.80
Vitality	7~35	23.47±4.38	4.69±0.87
**Sense of organizational support**	15~75	54.56±12.17	3.63±0.81

#### Correlation Analysis of Parenting Stress, Organizational Support, and Thriving at Work Among Clinical Nurses

##### The Mediating Role of Organizational Support Between Nurses’ Parenting Stress and Thriving at Work

To further analyze the relationship between organizational support, nurses’ parenting stress, and thriving at work, a model was created with thriving at work as the independent variable, organizational support as the intermediary variable, and nurses’ parenting stress as the dependent variable. Referring to the model correction index in the AMOS output results and the Pearson correlation analysis results (Table 3), the model was corrected layer by layer. The model’s fitting results are as follows: The chi-square to degree of freedom ratio (*χ* ^2^/df) is less than 5, the root mean square error of approximation (RMSEA) is less than 0.08, and the fitting indexes, such as the goodness of fit index (GFI), the adjusted goodness of fit index (AGFI), the Tucker-Lewis index (TLI), the normative fit index (NFI), the relative fit index (CFI), and the incremental fit index (IFI), are all satisfactory (Table 4).

**Table 3:** Correlation analysis of parenting stress, organizational support, and thriving at work among clinical nurses (N = 534) – China,2023.

Items	Work prosperity	Study	Vitality	Sense of organizational support	Parenting pressure on clinical nurses	Psychological burden	Physical and mental fatigue	Shift work	Working environment
Work prosperity	1								
Study	0.873***	1							
Vitality	0.894***	0.562***	1						
Sense of organizational support	0.469***	0.316***	0.505***	1					
Parenting pressure on clinical nurses	−0.233***	−0.153***	−0.255***	−0.289***	1				
psychological burden	−0.202***	−0.142***	−0.211***	−0.239***	0.796***	1			
Physical and mental fatigue	−0.202***	−0.149***	−0.205***	−0.239***	0.802***	0.515***	1		
Shift work	−0.178***	−0.095*	−0.215***	−0.254***	0.810***	0.469***	0.504***	1	
Working environment	−0.126**	−0.071	−0.149***	−0.144***	0.699***	0.417***	0.394***	0.572***	1

Note:

*P < 0.05

**P < 0.01

***P < 0.001

**Table 4:** Fitting indicators of the intermediary role model of organizational support for clinical nurses (N = 534) – China, 2023.

Items	Index value
Chi-square/Degrees of freedom (*χ* ^2^/df)	2.991
Root Mean Square Error of Approximation (RMSEA)	0.061
Goodness-of-Fit Index (GFI)	0.980
After-debugging Fit Index (AGFI)	0.954
Tuker-lewis Index (TLI)	0.958
Normative Fit Index (NFI)	0.965
Incremental Fit Index (IFI)	0.976
Comparative Fit Index (CFI)	0.976

**Figure 1:**
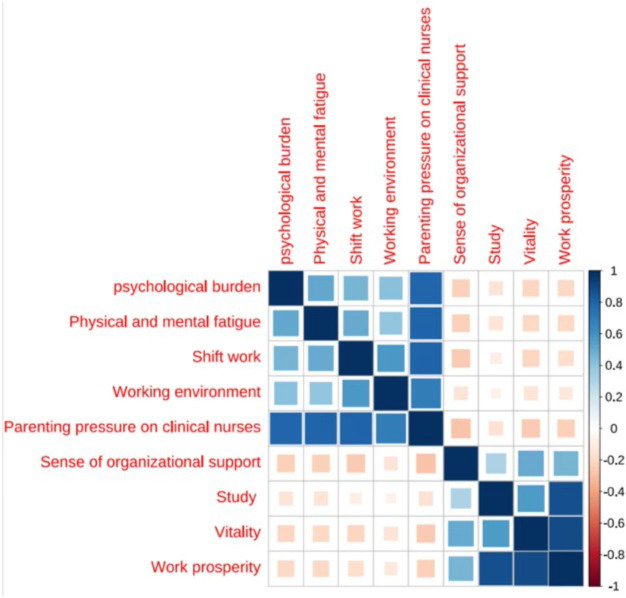
The correlation map shows the relationship between parenting stress, organizational support, and thriving.

Each path in the model, including parental stress of clinical nurses (β = −0.318) and organizational support (β = 0.487), is statistically significant (p < 0.05) (Figure 1).

#### Significance Test of the Mediator Effect

**Figure 2:**
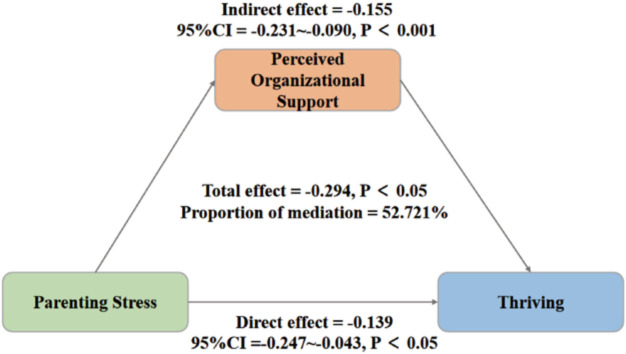
Mediation model of organizational support between parenting stress and thriving at work (N = 534).

Furthermore, the bootstrap method was used to test the mediating effect with 5,000 samples and a 95% confidence interval. The results show that the 95% confidence interval for the mediating effect of organizational support on parenting stress is (−0.231, −0.090) (Figure 2). Since the interval does not include 0, it is evident that organizational support partially mediates the relationship between parenting stress and thriving at work among clinical nurses. As shown in Table 5, the mediating effect accounts for 52.72% of the total effect.

**Table 5:** Intermediate effect test and bootstrap analysis results – China, 2023.

Path	Standardization coefficient	Standard error	95% confidence interval	
			Upper limit	Lower limit
Direct effect Parenting pressure of clinical nurses → thriving at work	−0.139	0.087	−0.247	−0.043
Indirect effect Parenting pressure of clinical nurses → organizational support → thriving at work	−0.155	0.108	−0.231	−0.090
Total effect	−0.294	0.108	−0.396	−0.186

## DISCUSSION

This study examines the relationship between parenting stress and thriving at work among nurses and whether organizational support mediates this relationship. We conducted a questionnaire survey of 534 clinical nurses using the Parenting Stress Scale for Clinical Nurses, the Thriving at Work Scale, and the Perceived Organizational Support Questionnaire. Our findings suggest that^(1)^ nurses’ total score on the sense of organizational support and thriving at work scale is at the middle level compared to the median value. The total score of nurses’ parenting stress was at an upper-middle level compared to the median value^(2)^. Second, nurse-perceived organizational support partially mediated the relationship between parenting stress and thriving at work.^(3)^ A sense of organizational support is positively correlated with thriving at work; the higher the sense of organizational support, the higher the thriving at work.

### Parenting Stress, Organizational Support, and Thriving at Work of Clinical Nurses

According to the theory of organizational support, employees are most willing to contribute to an organization if they feel that the organization cares about and values them. Employees who appreciate and support an organization are more likely to reach their full potential and excel^(26, 27)^. The results of this study show that nurses’ total score for their sense of organizational support is 3.63 ± 0.81, which is at a middle level compared to the median value and lower than ICU nurses’ results^(28)^, indicating that nurses’ sense of organizational support needs improvement. This may be related to the fact that the research subjects came from different departments, each with a different working environment, atmosphere, types of treatment, learning and training opportunities, and promotion policies. In this study, 188 nurses (47.2%) received extra care from their departments, such as flexible scheduling and vacation time. This indicates that departments need to strengthen the extra support they provide to nurses. Organizational support can neutralize the imbalance between nurses’ work and family lives, relieving the pressure and dilemmas they face in their career development and family relationships. This reduces their mental and psychological stress, improving the quality of nursing. Nursing managers should provide nurses with support beyond work. For example, they could regularly monitor nurses’ work status and adjust the frequency of night shifts to accommodate nurses’ physical conditions.

The total parenting stress score for nurses was 2.72 ± 0.47, which is an upper-middle level compared to the median value. This indicates that clinical nurses are facing higher levels of parenting stress. Among them, the shift work dimension score (2.89 ± 0.60) was the highest. The majority of nurses in this study are under the age of 35 (62.2%), and 66.9% of them participate in night shifts. Additionally, 74.5% of the nurses have given birth to one or more children, showing that many young nurses who work night shifts have children. Night shifts impose significant parenting challenges on nurses, potentially due to frequent overtime and night shifts, which can lead to an irregular lifestyle and insufficient rest. Nurses often have to “catch up on sleep” after work, leaving them with no extra energy or time to spend with their children, which results in greater parenting stress. Nursing managers should strictly implement the nursing leave system and pay attention to nurses with children who have no support. They should arrange regular day shifts or weekends for these nurses to give them enough time to spend with their children and reduce the pressure they face in raising their children. The results of this study also show that the total Thriving at Work score is 4.80 ± 0.74, which is a middle level compared to the median value and lower than the research results for clinical nurses^(29)^. This indicates that Thriving at Work among nurses needs urgent improvement.

Further analysis shows that the “vitality” dimension score (4.69 ± 0.87) is lower than the “learning” dimension score (4.91 ± 0.80). This indicates that nurses have a greater ability to acquire and apply knowledge and skills, but they need to strengthen their positive feelings of energy and enthusiasm at work to boost their self-confidence. This may be due to clinical nurses’ highly stressful work experience, passive sense of professional identity, and negative emotional state. Additionally, the results showed that 337 nurses (63.1%) worked over 40 hours per week and 463 nurses (86.7%) worked overtime more than once per week. This indicates that most clinical nurses are overloaded. These results suggest that nursing managers should reduce nurses’ workload by eliminating unnecessary examinations, training, and records to appropriately return nurses’ time to patient care. Simultaneously, we should pay more attention to nurses’ emotional well-being, understand their psychological state, provide them with more support at work, consider their needs, and address them promptly to stimulate their passion for work and enhance their professional growth.

### The Correlation Between Organizational Support, Nurses’ Parenting Stress, and Thriving at Work

The results of this study show that a sense of organizational support is positively correlated with thriving at work (r = 0.469, p < 0.001). In other words, the greater the sense of organizational support, the greater the thriving at work. This finding is similar to the results of other research^(30)^. One possible reason is that active organizational support can improve nurses’ sense of work self-efficacy, stimulate their inner sense of professional identity and belonging, strengthen their desire to learn new knowledge, and promote continuous individual growth and development. This, in turn, allows the organization to better achieve its goals and realize its “prosperous” development. Additionally, the parenting stress of clinical nurses is negatively correlated with thriving at work (r = **−**0.233, p < 0.001), indicating that higher parenting stress leads to lower thriving at work. One possible reason is that an overloaded nursing workload causes nurses to neglect caring for and educating their children, resulting in psychological pressure and guilt. When these negative emotions and pressures are brought to work, nurses’ learning initiative and vitality are reduced, thus affecting their work prosperity.

### The Mediating Role of Organizational Support Between Nurses’ Parenting Pressure and Thriving at Work

The results of this study showed that organizational support partially mediated the relationship between nurses’ parenting stress and thriving at work, accounting for 52.7% of the total effect. In other words, nurses’ parenting stress can directly affect thriving at work and indirectly mitigate its adverse effects and enhance thriving at work through the intermediary variable of organizational support. Parenting stress refers to the emotional state of parents experiencing anxiety, loss of social status, and physical fatigue when their parenting needs are not met due to influences from individuals, families, and society^(31)^. In clinical work, the parenting pressure nurses face hinders them from achieving their work goals and controlling their personal career development. In this situation, nurses tend to adopt negative coping strategies, which leads them to abandon efforts to improve themselves. This eventually results in a serious impact on their thriving at work. The embedded model of the job-prospering society holds that situational departmental characteristics significantly affect thriving at work^(17)^. Providing strong organizational support, cultivating a profound organizational culture, strengthening organizational fairness cognition, and fostering emotional commitment among members can significantly enhance nurses’ sense of belonging and make them feel cared for. This atmosphere relieves nurses’ parenting pressure, stimulates their enthusiasm for learning and work, and allows them to experience professional value and satisfaction, reducing negative emotions and achieving thriving at work. Suggestions for nursing managers: First, address the issue of parenting pressure faced by nurses and establish trust between managers and nurses so that nurses can promptly discuss issues and receive effective help when facing parenting challenges. Second, optimize the work system by offering valuable training courses and lectures, arranging flexible shifts for nursing mothers, and organizing group relaxation activities during holidays. Furthermore, managers should monitor the parenting stress levels of nursing parents in real time, provide reasonable and appropriate assistance and support, and strictly enforce relevant national policies to mitigate the negative effects of parenting issues on work efficiency and morale. These measures will help nurses better balance family and work, making it easier for them to achieve work-life balance.

Some hospitals have implemented targeted support measures in clinical practice to alleviate parenting-related stress among nurses. For instance, a “parenting nurse scheduling pool”^(32)^ has been established to prioritize day shifts and reduce the frequency of night shifts for nurses with young children. Pilot programs offering “parent-child leave” have been introduced, allowing nurses to take time off for important family moments with their children. Additionally, “nurse care stations”^(33)^ have been set up to provide psychological counseling, parenting seminars, and childcare services. To further reduce the burden, auxiliary nursing positions^(34)^ have been added to help manage the workload and minimize excessive overtime. These initiatives demonstrate the practical implementation of organizational care for nurses and provide a solid foundation for enhancing work motivation and improving nursing care quality.

## CONCLUSION

This study empirically examines the relationship between parenting stress and thriving at work among Chinese clinical nurses and identifies perceived organizational support as a key psychological mechanism underlying this association. Our findings reveal that higher levels of parenting stress are significantly associated with lower levels of work thriving, a critical indicator of positive psychological growth and professional vitality. Furthermore, perceived organizational support was found to partially mediate this relationship, accounting for over half (52.72%) of the total effect. These results suggest that, even when experiencing substantial family-related stress, strong organizational support can mitigate its negative effects and promote a sense of learning, energy, and engagement at work. These findings have important implications for nursing management and organizational policy. They underscore the need for healthcare institutions to recognize nurses as individuals navigating complex work-family dynamics, especially given China’s evolving fertility policies and the high proportion of young nurses with children in the workforce. Practical strategies, such as flexible scheduling, targeted emotional support, mentorship programs, and equitable promotion systems, may enhance nurses’ perception of organizational care. This would strengthen their resilience and promote sustained professional development. While this study is limited to a single center, its findings highlight the need for broader systemic attention to the intersection of family life and professional well-being in nursing. By fostering supportive work environments, nurse managers can mitigate the adverse effects of parenting stress and cultivate a culture of growth, belonging, and long-term retention within the nursing profession.

## Data Availability

The entire dataset supporting the results of this study was published in the article itself.
