# Supplementary material for: The relationship between parental stress and the mediating effect of organizational support perceived by nurses: a cross-sectional study
Source: Rev Esc Enferm USP. 2025 Dec 5;59:e20250162. doi: 10.1590/1980-220X-REEUSP-2025-0162en (PMC12703718; doi:10.1590/1980-220X-REEUSP-2025-0162en)
Supplement: Translation

# Translation: La relación entre el estrés parental y el efecto mediador del apoyo organizacional percibido por las enfermeras: un estudio transversal

**DOI:** 10.1590/1980-220X-REEUSP-2025-0162es

**Authors:** Na Liu, Lihua Niu, Qian Hu, Yan Wang, Donglian Zheng, Miao Yao, Guangli Mi

**Affiliations:** 1 Ningxia Medical University, General Hospital, Yinchuan, China.

**Keywords:** Cultura Organizacional, Enfermeros, Responsabilidad Parental, Negociación, Satisfacción en el Trabajo

## Abstract

**Objetivo:**

Examinar la relación entre el estrés parental y la prosperidad laboralentre enfermeros y enfermeras, y examinar si el apoyo organizacional media la asociación entre el estrés parental y el bienestar entre enfermeros y enfermeras.

**Métodos:**

Se realizó una encuesta mediante cuestionario a 534 enfermeros y enfermeras clínicos, utilizando la Escala de Estrés Parental para Enfermeros y Enfermeras Clínicos, la Escala de Bienestar en el Trabajo y el Cuestionario de Apoyo Organizacional Percibido.

**Resultados:**

Las puntuaciones totales del estrés parental de los enfermeros, del apoyo organizacional percibido por los enfermeros y de la Escala de Prosperidad en el Trabajo fueron (2,72 ± 0,47), (3,63 ± 0,81) y (4,80 ± 0,74), respectivamente. Se observó una correlación negativa significativa entre el estrés parental de la enfermería y el éxito en el trabajo (r1 = −0,233, P < 0,001) y el apoyo organizacional percibido por los enfermeros (r2 = −0,289, P < 0,001), así como una correlación positiva significativa entre el apoyo organizacional percibido por los enfermeros y el éxito en el trabajo (r3 = 0,469, P < 0,001). El apoyo organizacional percibido por los enfermeros mostró un efecto mediador parcial en la relación entre el estrés parental de los enfermeros y el éxito en el trabajo, representando el 52,72% del efecto total.

**Conclusión:**

Este estudio respalda el papel mediador del apoyo organizacional percibido por los enfermeros en la relación entre el estrés parental de los enfermeros y la prosperidad laboral. Deben estudiarse intervenciones para promover el apoyo organizacional percibido por los enfermeros con el fin de lograr el éxito en el trabajo de los enfermeros con estrés parental.

## INTRODUCCIÓN

A medida que se han desarrollado la psicología positiva y el comportamiento organizacional, un número cada vez mayor de investigadores ha estudiado formas de mejorar el entusiasmo de los enfermeros y fomentar el crecimiento mutuo de los enfermeros y las organizaciones a través de experiencias psicológicas positivas.

A pesar de la pesada carga de trabajo de la enfermería, algunas enfermeras pueden experimentar fatiga debido a su incapacidad para hacer frente a la presión laboral. Sin embargo, muchas enfermeras perseveran en sus funciones y experimentan un crecimiento positivo. Estudios anteriores han indicado que las enfermeras pueden experimentar emociones negativas, como el agotamiento y la fatiga, así como emociones positivas, como el crecimiento laboral a largo plazo^(1,2)^ y en su felicidad^(3,4)^.

El concepto de “prosperar” en el trabajo se propuso por primera vez en 2005^(5)^ y se define como un estado psicológico positivo en el que las personas experimentan aprendizaje y vitalidad en el trabajo. Las investigaciones demuestran que prosperar en el trabajo mejora la adaptabilidad^(6)^, mejora el rendimiento, reduce el agotamiento y promueve la salud^(7)^.

En China, las enfermeras son generalmente más jóvenes, con más del 60% de los profesionales de la enfermería menores de 35 años, y la mayoría de ellas son mujeres^(8)^. Las enfermeras en edad fértil desempeñan un papel fundamental en sus departamentos. Se enfrentan a presiones profesionales como la alta intensidad del trabajo, los horarios irregulares, el estrés mental a largo plazo y las presiones relacionadas con la promoción, la investigación científica y la enseñanza^(9,10)^. Mientras tanto, desde la implementación de la política de “dos hijos, tres hijos”, la atención se ha centrado en los problemas de fertilidad y los retos de la maternidad a los que se enfrentan las enfermeras en edad fértil. Las enfermeras que se reincorporan al trabajo después del parto a menudo se sienten poco familiarizadas con las prácticas clínicas y pueden experimentar una desconexión del trabajo clínico. Además, criar a los hijos requiere mucho tiempo y energía, lo que aumenta el estrés laboral. Muchas enfermeras afirman que “el cuidado de los hijos es estresante”^(11–13)^. El número de hijos se correlaciona positivamente con la rotación de personal de enfermería^(14)^, y la tasa de rotación de las enfermeras que han dado a luz a un segundo hijo alcanza el 62,73%^(15)^. Las enfermeras con responsabilidades parentales son especialmente susceptibles al estrés psicológico y físico, incluyendo ansiedad, fatiga y agotamiento profesional. Estos problemas se manifiestan a menudo a través de la privación del sueño y una mayor carga emocional. Estos retos pueden disminuir el compromiso con el trabajo y aumentar la probabilidad de rotación. Este agotamiento socava el bienestar de las enfermeras, compromete la calidad de la atención y aumenta el riesgo de errores clínicos, lo que pone en peligro la seguridad de los pacientes. Además, el estrés relacionado con la crianza de los hijos puede provocar un aumento del absentismo y las renuncias, lo que agrava la escasez de personal, intensifica la carga de trabajo del personal restante y, en última instancia, reduce la eficiencia operativa general. Estos problemas suponen obstáculos importantes para el funcionamiento estable y sostenible de las instituciones sanitarias. Por lo tanto, los directores de enfermería deben prestar mucha atención al estrés que experimentan las enfermeras clínicas en relación con la crianza de los hijos y reducir la rotación de personal.

Según el modelo de requisitos y recursos laborales, las personas necesitan recursos de otras personas u organizaciones para hacer frente al estrés^(16)^. El apoyo organizacional percibido (Perceived organizational support - POS) se refiere a si los empleados sienten que se valoran sus contribuciones y si se les cuida. Un alto sentido de apoyo organizacional puede proporcionar a los empleados un apoyo emocional positivo, mitigar los efectos negativos del estrés, ayudarles a mantener un estado activo, tanto física como psicológicamente, mejorar la prosperidad laboral^(17)^ y reducir su deseo de abandonar el trabajo^(18,19)^. Los estudiosos nacionales e internacionales se han centrado principalmente en el desarrollo profesional de las enfermeras y en la relación entre el desarrollo profesional, la creación de puestos de trabajo y la falta de atención de enfermería. Sin embargo, los mecanismos de mediación entre el estrés parental, la prosperidad laboral y el apoyo organizacional siguen sin estar claros.

Por lo tanto, este estudio tiene como objetivo examinar lo siguiente: 1) la situación del estrés parental, el apoyo organizacional y la prosperidad laboral de las enfermeras clínicas; 2) la relación entre el estrés parental, el apoyo organizacional percibido y la prosperidad laboral entre las enfermeras chinas; y 3) si el apoyo organizacional media la asociación entre el estrés parental y el bienestar en el trabajo.

## MÉTODO

### Consideraciones Éticas

Este estudio fue aprobado por el Hospital General de la Universidad Médica de Ningxia (KYLL-2024-0054). La portada del cuestionario explicaba claramente el propósito del estudio y garantizaba a los participantes su derecho al anonimato y la confidencialidad, así como su derecho a negarse a participar. Se obtuvo el consentimiento informado de todos los participantes.

### Diseño Del Estudio

Este estudio empleó un diseño cuantitativo y transversal. El informe se ajustó a las directrices STROBE (Strengthening the Reporting of Observational Studies in Epidemiology, Fortalecimiento de la notificación de estudios observacionales en epidemiología).

### Participantes

Los participantes en el estudio fueron reclutados en un hospital general de Ningxia, China, mediante un muestreo por conveniencia. Se invitó a participar en la encuesta a quienes cumplían los cuatro criterios de inclusión siguientes: 1) tener entre 22 y 45 años, 2) poseer un certificado de cualificación de enfermería, 3) ser pacientes y no familiares que acompañaban a pacientes, 4) estar registrados como enfermeros en activo, y 5) dar su consentimiento informado y participar voluntariamente. Se excluyó a las enfermeras en prácticas y en periodo de prueba, a las enfermeras avanzadas y a las enfermeras que no estaban trabajando, como las que estaban de baja por enfermedad, estudiando o realizando estudios de posgrado. Se contactó personalmente con las enfermeras que hablaban chino mandarín y trabajaban en el hospital para identificar a aquellas que cumplían los criterios de inclusión. Se les explicó el propósito de la encuesta y se les pidió que participaran según lo previsto.

La encuesta se llevó a cabo entre el 15 de diciembre de 2023 y el 19 de febrero de 2024. El cuestionario se administró a través de Wenjuanxing (https://www.wjx.cn/), la plataforma de cuestionarios en línea más popular de China. Las participantes rellenaron el cuestionario en línea. Solo se consideraron válidos los cuestionarios con respuestas a todas las preguntas, de acuerdo con nuestro criterio de validación predefinido. El 20 de agosto de 2022, descargamos los cuestionarios devueltos en formato Excel desde Wenjuanxing. Se devolvieron un total de 534 cuestionarios, lo que supuso una tasa de respuesta del 97,9% (534/545). Revisamos dos veces los cuestionarios devueltos y comprobamos que todos eran válidos.

## INSTRUMENTOS

### Variables Dependientes

Utilizamos la Escala de Prosperidad en el Trabajo (Thriving at Work Scale)^(20)^ para evaluar la prosperidad en el trabajo entre las enfermeras. Desarrollada en la Universidad de Georgetown en Estados Unidos en 2012, la escala ha sido traducida al chino^(21,22)^. La escala consta de diez ítems distribuidos en dos dimensiones: aprendizaje (cinco ítems) y vitalidad (cinco ítems). Empleamos una escala Likert de 7 puntos que va desde “totalmente en desacuerdo” a “totalmente de acuerdo”, con los ítems 4 y 8 puntuados de forma inversa. La puntuación total puede oscilar entre 10 y 70, y las puntuaciones más altas indican un mayor nivel de prosperidad laboral. El coeficiente α de Cronbach para esta escala fue de 0,822.

### Variable Independiente

Utilizamos la Escala de Estrés Parental (Parenting Stress Scale) para evaluar el estrés parental experimentado por las enfermeras. Desarrollada en Corea en 2021^(23)^, esta escala se tradujo posteriormente al chino^(24)^ y se utilizó para evaluar el estrés parental de las enfermeras. La escala consta de 19 ítems clasificados en cuatro dimensiones: carga psicológica (seis ítems), fatiga física y mental (cinco ítems), trabajo por turnos (cinco ítems) y entorno laboral (tres ítems). Se pidió a los encuestados que indicaran su nivel de acuerdo con cada ítem en una escala Likert de 4 puntos que iba desde “totalmente en desacuerdo” hasta “totalmente de acuerdo”. La puntuación total oscila entre 19 y 76 puntos. Una puntuación más alta indica un mayor estrés parental experimentado por las enfermeras. El coeficiente α de Cronbach total para esta escala es de 0,86, con coeficientes para las cuatro dimensiones que oscilan entre 0,81 y 0,86.

### Mediador

Utilizamos el Cuestionario de apoyo organizacional percibido (Perceived Organizational Support Questionnaire) para evaluar el nivel de apoyo organizacional experimentado por las enfermeras. Esta escala se desarrolló para medir el grado de apoyo organizacional a las enfermeras clínicas^(25)^. Se trata de una escala unidimensional que comprende 15 ítems. Utilizamos una escala Likert de 5 puntos que va desde “totalmente en desacuerdo” a “totalmente de acuerdo”, asignando puntuaciones del 1 al 5 en orden secuencial. Las puntuaciones totales oscilan entre 15 y 75, y las puntuaciones más altas indican un mayor sentido de apoyo organizacional. El índice de validez de contenido del cuestionario es de 0,940 y su coeficiente α de Cronbach es de 0,953.

### Covariables

Los análisis incluyeron las siguientes covariables: características demográficas y laborales, incluyendo sexo, edad, formación académica, título profesional, años de experiencia, método de contratación, departamento, puesto, número de guardias nocturnas al mes, horas de trabajo a la semana, frecuencia de horas extras a la semana y si el departamento proporcionaba cuidados adicionales.

### Análisis Estadístico

Describimos las características demográficas y laborales utilizando frecuencias, proporciones, medias y desviaciones estándar (DE). Se utilizaron pruebas de asimetría y curtosis para comprobar la normalidad de la distribución de las puntuaciones en las cuatro escalas. Se utilizó el análisis de correlación de Pearson para explorar las relaciones entre el estrés parental de las enfermeras, el apoyo organizacional y el bienestar en el trabajo. Se construyó un modelo de efectos mediadores del estrés parental de las enfermeras, el apoyo organizacional y el desarrollo profesional utilizando AMOS 23.0. Las macros de proceso de SPSS 4.0 evaluaron los efectos mediadores del apoyo organizacional en la relación entre el estrés parental y el desarrollo profesional. Se utilizó el método bootstrap percentil con corrección de la desviación para comprobar la significación del efecto mediador. Si el intervalo de confianza (IC) del 95% no incluía el cero, se consideraba que el efecto mediador era significativo. Las características demográficas y relacionadas con el trabajo se incluyeron como covariables en este modelo. La significación estadística se determinó con un valor p inferior a 0,05 (bilateral).

### Análisis De Correlación Entre El Estrés Parental, el Apoyo Organizacional y el Bienestar Laboral Entre Enfermeras Clínicas

## RESULTADO

### Estadísticas Descritivas

#### Caracterización Sociodemográfica de Las Enfermeras

Este estudio examinó las características sociodemográficas de 534 enfermeras en China en 2023 (Tabla 1). Los resultados revelaron que la fuerza laboral de enfermería era predominantemente femenina (94,0%) y que el grupo de edad más numeroso era el de 35 a 45 años (37,8%). En cuanto a la educación, la mayoría tenía una licenciatura (79,8%), mientras que solo el 2,2% tenía una maestría o un título superior. Los títulos profesionales eran principalmente junior (55,8%) e intermedios (37,6%), y la mayoría de las enfermeras eran personal no permanente (75,5%). La experiencia laboral se distribuía de manera relativamente uniforme, con la mayor proporción entre seis y 15 años de servicio (53,7% en total). La mayoría (76,0%) no ocupaba puestos directivos, y los departamentos más comunes eran medicina interna (31,3%) y cirugía (20,0%). En cuanto a los turnos de noche, el 33.1% de las enfermeras no los realizaba, mientras que el 30.5% trabajaba entre cinco y nueve al mes. En cuanto a las horas de trabajo, el 63.1% trabajaba más de 40 horas a la semana y el 41.0% a veces hacía horas extras.

**Tabla 1:** Caracterización sociodemográfica de las enfermeras (n = 534) – China, 2023.

Elementos	Categoría	Cantidad	Porcentaje (%)
Género	Masculino	32	6,0
	Mujer	502	94,0
Edad (años)	22~29	162	30,4
	30~34	170	31,8
	35~45	202	37,8
Titulación académica oficial	Título universitario o inferior	96	19
	Licenciatura	426	79,8
	Estudiantes de posgrado y superiores	12	2,2
Título profesional	Primario	298	55,8
	Rango medio	201	37,6
	Subdirector y superiores	35	6,6
Modalidad de empleo	Sin editar	403	75,5
	Edición oficial	131	24,5
Vida laboral	≤5	131	24,5
	6~10	146	27,3
	11~15	141	26,4
	16~20	58	10,9
	>20	58	10,9
Posición	Sin	406	76,0
	Líder docente o líder responsable	89	16,7
	Jefa de enfermería	39	7,3
Departamento	Medicina interna	167	31,3
	Cirugía	107	20,0
	Ginecología y obstetricia	26	4,9
	Pediatría	35	6,6
	Unidad de cuidados intensivos	29	5,4
	Servicio de urgencias	62	11,6
	Quirófano	28	5,2
	Otros	80	15,0
Guardias nocturnas al mes	Sin guardias nocturnas	177	33,1
	≤4	96	18,0
	5~9	163	30,5
	≥10	98	18,4
Horas de trabajo semanales (horas)	<40	75	14,0
	40	122	22,8
	>40	337	63,1
Frecuencia de horas extras por semana	Casi nunca	71	13,3
	Rara vez	151	28,3
	A veces	219	41,0
	A menudo	93	17,4

#### Situación Actual del Estrés Parental, el Apoyo Organizativo y El Bienestar Laboral Entre Las Enfermeras Clínicas

Los resultados revelaron una puntuación global de estrés parental de 2,72 ± 0,47 entre las enfermeras clínicas (Tabla 2). La puntuación más alta en la subescala fue la del trabajo por guardias (2,89 ± 0,60), seguida de la del ambiente de trabajo (2,82 ± 0,63), la carga psicológica (2,64 ± 0,54) y la fatiga física y mental (2,59 ± 0,65). En cuanto al desarrollo en el trabajo, la puntuación total fue de 4,80 ± 0,74; la dimensión “Estudio” obtuvo una puntuación inferior a la dimensión “Vitalidad”. El apoyo organizativo percibido obtuvo una puntuación de 3,63 ± 0,81.

**Tabla 2:** Situación actual del estrés parental, el apoyo organizativo y el desarrollo personal entre las enfermeras clínicas (N = 534) – China en 2023.

Ítems	Rango de puntuación	Puntuación agregada	Entrada media
**Presión parental de las enfermeras clínicas**	19~76	51,75 ± 9,00	2,72 ± 0,47
Carga psicológica	4~24	15,83 ± 3,27	2,64 ± 0,54
Fatiga física y mental	5~20	12,98 ± 3,25	2,59 ± 0,65
Trabajo por guardias	5~20	14,45 ± 3,02	2,89 ± 0,60
Ambiente laboral	3~12	8,47 ± 1,91	2,82 ± 0,63
**Prosperidad laboral**	14~70	48,06 ± 7,43	4,80 ± 0,74
Estudio	7~35	24,59 ± 4,02	4,91 ± 0,80
Vitalidad	7~35	23,47 ± 4,38	4,69 ± 0,87
**Sensación de apoyo organizativo**	15~75	54,56 ± 12,17	3,63 ± 0,81

#### El Papel Mediador Del Apoyo Organizacional Entre el Estrés Parental de Las Enfermeras y el Desarrollo en el Trabajo

Para analizar más a fondo la relación entre el apoyo organizacional, el estrés parental de las enfermeras y el desarrollo en el trabajo, se creó un modelo con el desarrollo en el trabajo como variable independiente, el apoyo organizacional como variable intermediaria y el estrés parental de las enfermeras como variable dependiente. Teniendo en cuenta el índice de corrección del modelo en los resultados de AMOS y los resultados del análisis de correlación de Pearson (Tabla 3), el modelo se corrigió capa por capa. Los resultados de ajuste del modelo son los siguientes: la relación entre la chi cuadrada y el grado de libertad (*χ* ^2^/df) es inferior a 5, el error cuadrático medio de aproximación (RMSEA) es inferior a 0,08 y los índices de ajuste, como el índice de bondad de ajuste (GFI), el índice de bondad de ajuste ajustado (AGFI), el índice de Tucker-Lewis (TLI), el índice de ajuste normativo (NFI), el índice de ajuste relativo (CFI) y el índice de ajuste incremental (IFI) son satisfactorios (Tabla 4).

**Table 3:** Análisis de correlación entre el estrés parental, el apoyo organizacional y el bienestar laboral entre enfermeras clínicas (N = 534) – China, 2023.

Elementos	Prosperidad laboral	Estudio	Vitalidad	Sensación de apoyo organizacional	Presión parental sobre las enfermeras clínicas	Carga psicológica	Fatiga física y mental	Trabajo por guardias	Ambiente laboral
Prosperidad laboral	1								
Estudio	0,873***	1							
Vitalidad	0,894***	0,562***	1						
Sensación de apoyo organizativo	0,469	0,316	0,505	1					
Presión parental sobre las enfermeras clínicas	−0,233***	−0,153***	−0,255***	−0,289	1				
Carga psicológica	−0,202***	−0,142***	−0,211***	−0,239***	0,796***	1			
Fatiga física y mental	−0,202***	−0,149***	−0,205***	−0,239***	0,802	0,515***	1		
Trabajo por turnos	−0,178***	−0,095*	−0,215***	−0,254***	0,810***	0,469	0,504***	1	
Ambiente de trabajo	−0,126**	−0,071	−0,149***	−0,144***	0,699	0,417***	0,394	0,572***	1

**Tabla 4:** Indicadores de ajuste del modelo de papel intermediario del apoyo organizacional para enfermeras clínicas (N = 534) – China, 2023.

Elementos	Valor del índice
Chi-cuadrado/Grados de libertad (*χ* ^2^ /df)	2,991
Error cuadrático medio de aproximación (RMSEA)	0,061
Índice de bondad de ajuste (GFI)	0,980
Índice de ajuste tras depuración (AGFI)	0,954
Índice de Tuker-Lewis (TLI)	0,958
Índice de ajuste normativo (NFI)	0,965
Índice de ajuste incremental (IFI)	0,976
Índice de ajuste comparativo (CFI)	0,976

Cada ruta del modelo, incluido el estrés parental de las enfermeras clínicas (β = −0,318) y el apoyo organizacional (β = 0,487), es estadísticamente significativa (p < 0,05) (Figura 1).

**Figura 1:** El mapa de correlación muestra la relación entre el estrés parental, el apoyo organizacional y el bienestar.

#### Prueba de Significación Del Efecto Mediador

Además, se utilizó el método bootstrap para probar el efecto mediador con 5000 muestras y un intervalo de confianza del 95%. Los resultados muestran que el intervalo de confianza del 95% para el efecto mediador del apoyo organizacional sobre el estrés parental es (−0,231, −0,090) (Figura 2). Dado que el intervalo no incluye el 0, es evidente que el apoyo organizacional media parcialmente la relación entre el estrés parental y el desarrollo profesional de las enfermeras clínicas. Como se muestra en la Tabla 5, el efecto mediador representa el 52,72% del efecto total.

**Figura 2:** Modelo de mediación del apoyo organizacional entre el estrés parental y la prosperidad en el trabajo (N = 534).

**Tabla 5:** Prueba de efecto intermedio y resultados del análisis bootstrap – China, 2023.

Ruta	Coeficiente de estandarización	Error estándar	Intervalo de confianza del 95	
			Límite superior	Límite inferior
Efecto directo Presión parental de las enfermeras clínicas → prosperidad en el trabajo	−0,139	0,087	−0,247	−0,043
Efecto indirecto Presión parental de las enfermeras clínicas → apoyo organizativo → prosperidad en el trabajo	−0,155	0,108	−0,231	−0,090
Efecto total	−0,294	0,108	−0,396	−0,186

## DISCUSIÓN

Este estudio examina la relación entre el estrés parental y el bienestar laboral entre las enfermeras, y si el apoyo organizacional media en esta relación. Realizamos una encuesta a 534 enfermeras clínicas utilizando la Escala de Estrés Parental para Enfermeras Clínicas, la Escala de Prosperidad Laboral y el Cuestionario de Apoyo Organizacional Percibido. Nuestros hallazgos sugieren que^(1)^ la puntuación total de las enfermeras en la escala de sensación de apoyo organizacional y prosperidad en el trabajo se encuentra en un nivel medio en comparación con el valor mediano. La puntuación total del estrés parental de las enfermeras se situó en un nivel medio-alto en comparación con el valor mediano^(2)^. En segundo lugar, el apoyo organizacional percibido por las enfermeras mediaba parcialmente la relación entre el estrés parental y el desarrollo en el trabajo. ^(3)^ La sensación de apoyo organizacional se correlaciona positivamente con el desarrollo en el trabajo; cuanto mayor es la sensación de apoyo organizacional, mayor es el desarrollo en el trabajo.

### Presión Parental, Apoyo Organizacional y Desarrollo en el Trabajo de Las Enfermeras Clínicas

Según la teoría del apoyo organizacional, los empleados están más dispuestos a contribuir a una organización si sienten que esta se preocupa por ellos y los valora. Los empleados que aprecian y apoyan a una organización son más propensos a alcanzar su máximo potencial y a sobresalir^(26,27)^. Los resultados de este estudio muestran que la puntuación total de las enfermeras en cuanto a su sensación de apoyo organizacional es de 3,63 ± 0,81, lo que se sitúa en un nivel medio en comparación con el valor medio y es inferior a los resultados de las enfermeras de la UCI^(28)^, lo que indica que la sensación de apoyo organizacional de las enfermeras necesita mejorar. Esto puede estar relacionado con el hecho de que los sujetos de la investigación procedían de diferentes departamentos, cada uno con un entorno de trabajo, un ambiente, tipos de tratamiento, oportunidades de aprendizaje y formación y políticas de promoción diferentes. En este estudio, 188 enfermeras (47,2%) recibieron atención adicional de sus departamentos, como horarios flexibles y vacaciones. Esto indica que los departamentos deben reforzar el apoyo adicional que prestan a las enfermeras. El apoyo organizativo puede neutralizar el desequilibrio entre la vida laboral y familiar de las enfermeras, aliviando la presión y los dilemas a los que se enfrentan en el desarrollo de su carrera y en sus relaciones familiares. Esto reduce su estrés mental y psicológico, mejorando la calidad de la atención de enfermería. Los directores de enfermería deben proporcionar a las enfermeras un apoyo que vaya más allá del trabajo. Por ejemplo, podrían supervisar regularmente la situación laboral de las enfermeras y ajustar la frecuencia de los turnos de noche para adaptarse a sus condiciones físicas.

La puntuación total de estrés parental de las enfermeras fue de 2,72 ± 0,47, lo que supone un nivel medio-alto en comparación con el valor medio. Esto indica que las enfermeras clínicas se enfrentan a niveles más altos de estrés parental. Entre ellas, la puntuación de la dimensión del trabajo por turnos (2,89 ± 0,60) fue la más alta. La mayoría de las enfermeras de este estudio tienen menos de 35 años (62,2%) y el 66,9% de ellas participan en guardias nocturnas. Además, el 74,5% de las enfermeras han dado a luz a uno o más hijos, lo que demuestra que muchas enfermeras jóvenes que trabajan en guardias nocturnas tienen hijos. Los guardias nocturnas suponen un reto importante para las enfermeras en lo que respecta a la crianza de los hijos, posiblemente debido a las frecuentes horas extras y los guardias nocturnas, que pueden conducir a un estilo de vida irregular y a un descanso insuficiente. Las enfermeras a menudo tienen que “recuperar el sueño” después del trabajo, lo que les deja sin energía ni tiempo extra para pasar con sus hijos, lo que se traduce en un mayor estrés parental. Los directores de enfermería deben aplicar estrictamente el sistema de permisos de maternidad y prestar atención a las enfermeras con hijos que no cuentan con apoyo. Deben organizar turnos diurnos o fines de semana regulares para estas enfermeras, a fin de que tengan tiempo suficiente para pasar con sus hijos y reducir la presión a la que se enfrentan en la crianza de sus hijos. Los resultados de este estudio también muestran que la puntuación total de “Prosperar en el trabajo” es de 4,80 ± 0,74, lo que supone un nivel medio en comparación con el valor mediano y es inferior a los resultados de la investigación sobre enfermeras clínicas^(29)^. Esto indica que es necesario mejorar urgentemente la “Prosperidad en el trabajo” entre las enfermeras.

Un análisis más detallado muestra que la puntuación de la dimensión “vitalidad” (4,69 ± 0,87) es inferior a la de la dimensión “aprendizaje” (4,91 ± 0,80). Esto indica que las enfermeras tienen una mayor capacidad para adquirir y aplicar conocimientos y habilidades, pero necesitan reforzar sus sentimientos positivos de energía y entusiasmo en el trabajo para aumentar su confianza en sí mismas. Esto puede deberse a la experiencia laboral altamente estresante de las enfermeras clínicas, su sentido pasivo de identidad profesional y su estado emocional negativo. Además, los resultados mostraron que 337 enfermeras (63,1%) trabajaban más de 40 horas a la semana y 463 enfermeras (86,7%) hacían horas extras más de una vez a la semana. Esto indica que la mayoría de las enfermeras clínicas están sobrecargadas. Estos resultados sugieren que los directores de enfermería deberían reducir la carga de trabajo de las enfermeras eliminando exámenes, formaciones y registros innecesarios para que las enfermeras puedan dedicar más tiempo al cuidado de los pacientes. Al mismo tiempo, deberíamos prestar más atención al bienestar emocional de las enfermeras, comprender su estado psicológico, proporcionarles más apoyo en el trabajo, tener en cuenta sus necesidades y atenderlas con prontitud para estimular su pasión por el trabajo y mejorar su crecimiento profesional.

### La Correlación Entre el Apoyo Organizativo, el Estrés Parental de Las Enfermeras y El Éxito en el Trabajo

Los resultados de este estudio muestran que la sensación de apoyo organizativo está correlacionada positivamente con la prosperidad laboral (r = 0,469, p < 0,001). En otras palabras, cuanto mayor es la sensación de apoyo organizativo, mayor es el éxito en el trabajo. Este hallazgo es similar a los resultados de otras investigaciones^(30)^. Una posible razón es que el apoyo activo de la organización puede mejorar la sensación de autoeficacia laboral de las enfermeras, estimular su sentido interno de identidad profesional y pertenencia, fortalecer su deseo de aprender nuevos conocimientos y promover el crecimiento y el desarrollo individual continuo. Esto, a su vez, permite a la organización alcanzar mejor sus objetivos y realizar su desarrollo “próspero”. Además, el estrés parental de las enfermeras clínicas se correlaciona negativamente con la prosperidad laboral (r = −0,233, p < 0,001), lo que indica que un mayor estrés parental conduce a un menor bienestar en el trabajo. Una posible razón es que una carga de trabajo excesiva en enfermería hace que las enfermeras descuiden el cuidado y la educación de sus hijos, lo que les provoca presión psicológica y culpa. Cuando estas emociones y presiones negativas se trasladan al trabajo, la iniciativa de aprendizaje y la vitalidad de las enfermeras se reducen, lo que afecta a su prosperidad laboral.

### El Papel Mediador Del Apoyo Organizativo Entre ea Presión Parental de Las Enfermeras y el Desarrollo en el Trabajo

Los resultados de este estudio mostraron que el apoyo organizativo mediaba parcialmente la relación entre el estrés parental de las enfermeras y el desarrollo en el trabajo, representando el 52,7% del efecto total. En otras palabras, el estrés parental de las enfermeras puede afectar directamente al rendimiento en el trabajo e indirectamente mitigar sus efectos adversos y mejorar el rendimiento en el trabajo a través de la variable intermediaria del apoyo organizacional. El estrés parental se refiere al estado emocional de los padres que experimentan ansiedad, pérdida de estatus social y fatiga física cuando sus necesidades parentales no se satisfacen debido a influencias de individuos, familias y la sociedad^(31)^. En el trabajo clínico, la presión parental a la que se enfrentan las enfermeras les impide alcanzar sus objetivos laborales y controlar su desarrollo profesional personal. En esta situación, las enfermeras tienden a adoptar estrategias de afrontamiento negativas, lo que les lleva a abandonar sus esfuerzos por mejorar. Esto acaba teniendo un grave impacto en su prosperidad en el trabajo. El modelo integrado de la sociedad próspera en el trabajo sostiene que las características situacionales de los departamentos afectan significativamente a la prosperidad en el trabajo^(17)^. Proporcionar un fuerte apoyo organizativo, cultivar una cultura organizativa profunda, reforzar la percepción de la equidad organizativa y fomentar el compromiso emocional entre los miembros puede mejorar significativamente el sentido de pertenencia de las enfermeras y hacerlas sentir cuidadas. Este ambiente alivia la presión parental de las enfermeras, estimula su entusiasmo por el aprendizaje y el trabajo, y les permite experimentar el valor y la satisfacción profesionales, reduciendo las emociones negativas y logrando prosperar en el trabajo. Sugerencias para los directores de enfermería: En primer lugar, abordar la cuestión de la presión parental a la que se enfrentan las enfermeras y establecer la confianza entre los directores y las enfermeras, de modo que estas últimas puedan discutir rápidamente los problemas y recibir ayuda eficaz cuando se enfrenten a retos parentales. En segundo lugar, optimizar el sistema de trabajo ofreciendo cursos de formación y conferencias valiosos, organizando turnos flexibles para las madres lactantes y organizando actividades de relajación en grupo durante las vacaciones. Además, los directores deben supervisar en tiempo real los niveles de estrés parental de los padres que trabajan en enfermería, proporcionarles una ayuda y un apoyo razonables y adecuados, y aplicar estrictamente las políticas nacionales pertinentes para mitigar los efectos negativos de los problemas de crianza en la eficiencia y la moral en el trabajo. Estas medidas ayudarán a las enfermeras a conciliar mejor la vida familiar y laboral, facilitándoles el equilibrio entre el trabajo y la vida personal.

Algunos hospitales han aplicado medidas de apoyo específicas en la práctica clínica para aliviar el estrés relacionado con la crianza de los hijos entre las enfermeras. Por ejemplo, se ha creado una “bolsa de guardias para enfermeras con hijos”^(32)^ para dar prioridad a los turnos diurnos y reducir la frecuencia de las guardias nocturnas de las enfermeras con hijos pequeños. Se han introducido programas piloto que ofrecen “licencia parental”, lo que permite a las enfermeras tomarse tiempo libre para momentos familiares importantes con sus hijos. Además, se han creado “estaciones de atención a enfermeras”^(33)^ para proporcionar asesoramiento psicológico, seminarios sobre crianza y servicios de cuidado infantil. Para reducir aún más la carga, se han añadido puestos de enfermería auxiliar^(34)^ para ayudar a gestionar la carga de trabajo y minimizar las horas extras excesivas. Estas iniciativas demuestran la aplicación práctica del cuidado organizativo de las enfermeras y proporcionan una base sólida para mejorar la motivación laboral y la calidad de la atención de enfermería.

## CONCLUSIÓN

Este estudio examina empíricamente la relación entre el estrés parental y la prosperidad laboral entre las enfermeras clínicas chinas e identifica el apoyo organizativo percibido como un mecanismo psicológico clave que subyace a esta asociación. Nuestros hallazgos revelan que los niveles más altos de estrés parental se asocian significativamente con niveles más bajos de prosperidad laboral, un indicador crítico del crecimiento psicológico positivo y la vitalidad profesional. Además, se descubrió que el apoyo organizacional percibido mediaba parcialmente esta relación, representando más de la mitad (52,72%) del efecto total. Estos resultados sugieren que, incluso cuando se experimenta un estrés familiar considerable, un fuerte apoyo organizacional puede mitigar sus efectos negativos y promover una sensación de aprendizaje, energía y compromiso en el trabajo. Estos hallazgos tienen importantes implicaciones para la gestión de la enfermería y la política organizativa. Subrayan la necesidad de que las instituciones sanitarias reconozcan a las enfermeras como personas que se enfrentan a una dinámica compleja entre el trabajo y la familia, especialmente teniendo en cuenta la evolución de las políticas de fertilidad en China y la alta proporción de enfermeras jóvenes con hijos en la población activa. Las estrategias prácticas, como la flexibilidad horaria, el apoyo emocional específico, los programas de mentoría y los sistemas de promoción equitativos, pueden mejorar la percepción que tienen las enfermeras del cuidado organizativo. Esto reforzaría su resiliencia y promovería un desarrollo profesional sostenido. Aunque este estudio se limita a un solo centro, sus conclusiones ponen de relieve la necesidad de prestar una atención sistémica más amplia a la intersección entre la vida familiar y el bienestar profesional en la enfermería. Al fomentar entornos de trabajo favorables, los directores de enfermería pueden mitigar los efectos adversos del estrés parental y cultivar una cultura de crecimiento, pertenencia y retención a largo plazo dentro de la profesión de enfermería.

## Data Availability

Todo el conjunto de datos que apoya los resultados de este estudio fue publicado en el propio artículo.
